# CD70 contributes to age-associated T cell defects and overwhelming inflammatory responses

**DOI:** 10.18632/aging.103368

**Published:** 2020-06-19

**Authors:** Di Wang, Juan Du, Yangzi Song, Beibei Wang, Rui Song, Yu Hao, Yongqin Zeng, Jiang Xiao, Hong Zheng, Hui Zeng, Hongxin Zhao, Yaxian Kong

**Affiliations:** 1 Beijing Key Laboratory of Emerging Infectious Diseases, Institute of Infectious Diseases, Beijing Ditan Hospital, Capital Medical University, Beijing 100015, China; 2 Clinical and Research Center of Infectious Diseases, Beijing Ditan Hospital, Capital Medical University, Beijing 100015, China; 3 Penn State Cancer Institute, Penn State University College of Medicine, Hershey, PA 17033, USA

**Keywords:** CD70, T cell aging, immunosenescence, overwhelming inflammatory responses, co-inhibitory molecules

## Abstract

Aging is associated with immune dysregulation, especially T cell disorders, which result in increased susceptibility to various diseases. Previous studies have shown that loss of co-stimulatory receptors or accumulation of co-inhibitory molecules play important roles in T cell aging. In the present study, CD70, which was generally regarded as a costimulatory molecule, was found to be upregulated on CD4^+^ and CD8^+^ T cells of elderly individuals. Aged CD70^+^ T cells displayed a phenotype of over-activation, and expressed enhanced levels of numerous inhibitory receptors including PD-1, 2B4 and LAG-3. CD70^+^ T cells from elderly individuals exhibited increased susceptibility to apoptosis and high levels of inflammatory cytokines. Importantly, the functional dysregulation of CD70^+^ T cells associated with aging was reversed by blocking CD70. Collectively, this study demonstrated CD70 as a prominent regulator involved in immunosenescence, which led to defects and overwhelming inflammatory responses of T cells during aging. These findings provide a strong rationale for targeting CD70 to prevent dysregulation related to immunosenescence.

## INTRODUCTION

Aging is accompanied by dysregulated immune responses that result in high susceptibility to various diseases [1–3]. It is characterized as immunosenescence, which involves a gradual deterioration of immunity as well as enhanced inflammatory responses [4, 5]. In particular, T cell aging is considered to be a prominent contributor to age-associated-immune dysregulation [6, 7].

Co-stimulatory and co-inhibitory molecules are crucial for regulating T cell activation, differentiation, effector function and survival [8]. Loss of some co-stimulatory receptors, such as CD28 and CD27, is one of the most consistent immunological markers of T cell aging [9, 10]. Co-inhibitory molecules also play important roles in T cell aging. In murine models as well as in humans, programmed death-1 (PD-1), T-cell immunoglobulin domain and mucin domain 3 (TIM-3), lymphocyte-activation gene 3 (LAG-3), cytotoxic T-lymphocyte-associated protein 4 (CTLA-4), and tyrosine-based inhibitory motif (ITIM) domain (TIGIT) were found to be upregulated during aging [11–14]. These findings suggest that suppressive pathways contribute to immunosenescence. However, the mechanism that regulates enhanced inflammatory responses of T cells has not been established. Additionally, the effects of newly identified co-signaling molecules during aging needs to be investigated.

CD70 is the sole ligand for co-stimulatory receptor CD27 [15]. Thus, it was generally considered as a stimulatory molecule. It is expressed on antigen presenting cells (APCs), epithelial cells, mature dendritic cells, and many types of tumor cells [16–20]. However, recent studies indicated T cell-derived CD70 as an inhibitory molecule in patients with B-cell non-Hodgkin’s lymphoma and murine models of inflammatory bowel disease or allogeneic graft-versus-host disease [21, 22]. Herein, we assessed the role of CD70 in T cell immunosenescence using blood samples from healthy individuals. Overall, this study suggests that CD70 upregulation is an important process associated with T cell aging, which leads to defects and overwhelming inflammatory responses of T cells.

## RESULTS

### Age-related CD70 accumulation in CD4^+^ and CD8^+^ T cells

**Figure 1:**
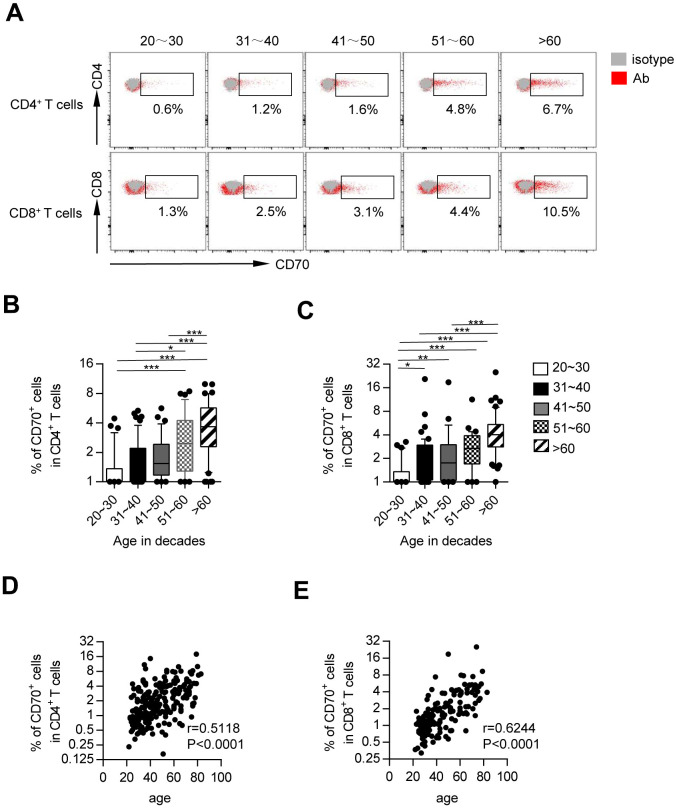
**CD70-expressing T cells accumulate with age.** Flow cytometry analysis of CD70 expression on PBMCs from healthy controls of different ages. (**A**) Representative flow cytometric plots show the expression of CD70 gated on CD4^+^ and CD8^+^ T cells from five healthy donors in different age groups. (**B**–**C**) Box plots of the frequencies of CD70^+^ cells among CD4^+^ and CD8^+^ T cells from healthy donors in different age groups (n = 34-56 in each group). Values given are the median frequencies ± the interquartile range and 10 and 90 percentile whiskers. The *p*-values were obtained by Kruskal-Wallis test followed by Dunn’s multiple comparisons test. (**D**–**E**) Correlation analysis of age and surface CD70 expression on CD4^+^ T cells (**D**) and CD8^+^ T cells (**E**) from all healthy individuals. Spearman’s non-parametric test was used to test for correlations. *p < 0.05, **p < 0.01, ***p < 0.001.

To investigate the potential role of CD70 signaling in T-cell aging, we examined the expression of CD70 on T cells from 217 healthy adults using flow cytometry (Table 1). The results showed that CD70-expressing CD4^+^ and CD8^+^ T cells accumulated with aging. The frequencies of CD70^+^ fractions among CD4^+^ and CD8^+^ T cells from the elderly (61-80 years) were significantly higher than those from young (21-30 and 31-40 years) and middle-aged individuals (41-50 and 51-60 years; Figure 1A–1C). Additionally, middle-aged individuals showed higher expression of CD70 as compared to young individuals. Correlation analysis showed a strong correlation of CD70 expression on CD4^+^ and CD8^+^ T cells with age (CD4: r = 0.5118, *p* < 0.0001; CD8: r = 0.6244, *p* < 0.0001; Figure 1D–1E).

**Table 1:** Characteristics of subjects in this study.

**parameters**	**Total (n=217)**	**20~30 (n=39)**	**31~40 (n=56)**	**41~50 (n=36)**	**51~60 (n=34)**	**61~80 (n=52)**	***P* Value**
**Gender**							
Male	94	18	29	14	14	19	0.5433
Female	123	21	27	22	20	33	
**Age, years**							
Median	47	27	36	45	55	70	0.2071
IQR	34-60	25-29	34-37	43-48	53-59	64-74	

A total of 217 healthy adults were recruited, including 94 males and 123 females. Their median age was 45, and 34-60 adults were in every group. The Chi-square test demonstrated that the gender was balanced among all the groups (*P* = 0.5433). Age was described by median and interquartile rage (IQR) and analyzed using Kruskal-Wallis test.

### CD70 was up-regulated on each subset of circulating T cells during aging

**Figure 2:**
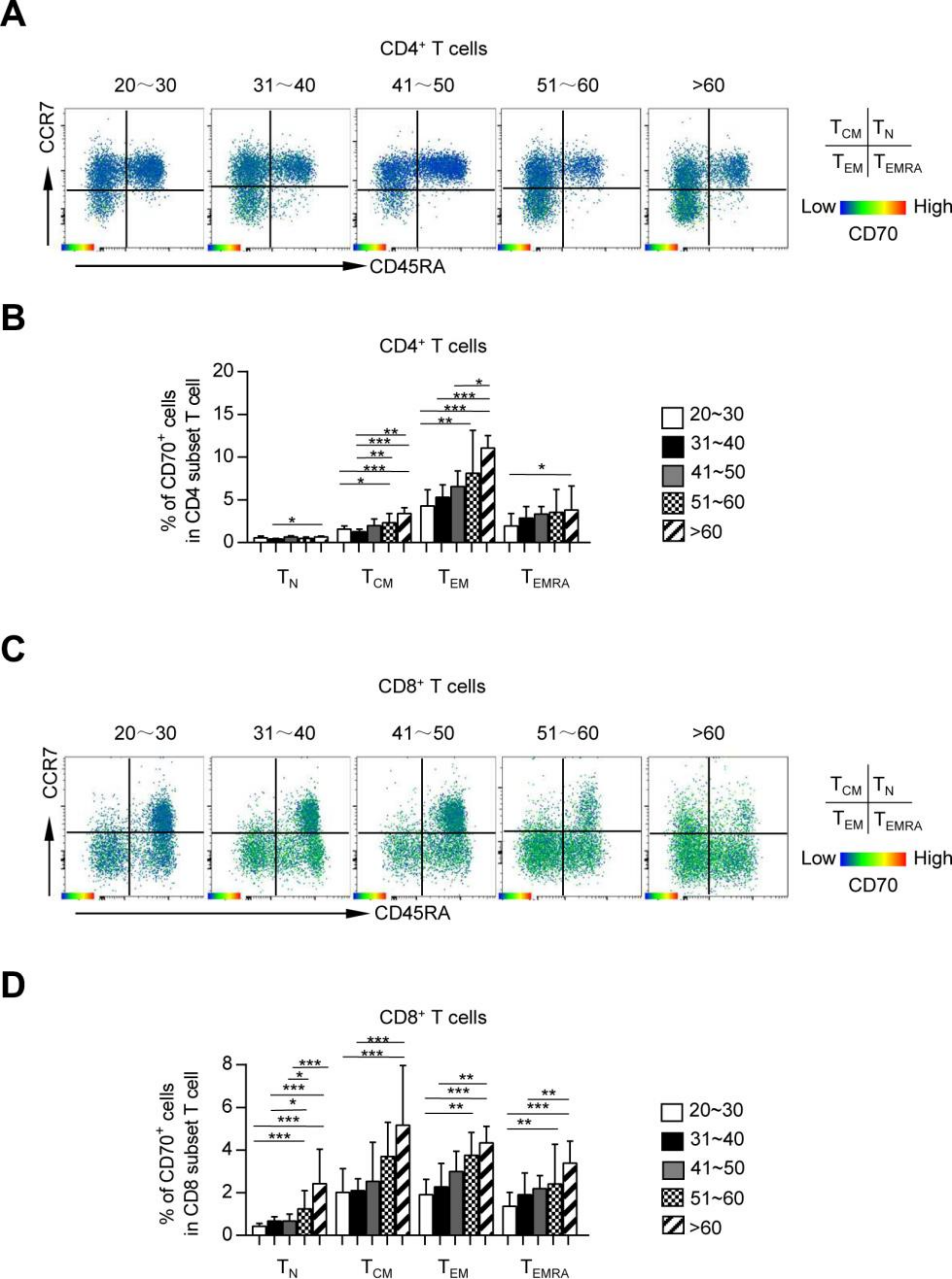
**CD70 is preferentially expressed on memory CD4^+^ and CD8^+^ T cells.** Expression of CD70 on each subset (T_N_, T_CM_, T_EM_, and T_EMRA_) of CD4^+^ and CD8^+^ T cells. Representative flow data (**A**, **C**) and box plots (**B**, **D**) of the percentage of CD70 expression on each subset of CD4^+^ (**A**–**B**) and CD8^+^ (**C**–**D**) T cells from five different age groups (n = 34-56 in each group). Data are shown as the median ± 95% confidence interval (CI). The *p*-values were obtained by Kruskal-Wallis test followed by Dunn’s multiple comparisons test. *p < 0.05, **p < 0.01, ***p < 0.001.

Since previous studies including ours reported an expansion of antigen-experienced T cells in the elderly population [14, 23], we investigated whether heterogeneous T cell subsets expressed different levels of CD70 in this study. Based on the expression of CD45RA and CCR7, the T cells were divided into four subsets: naïve T cells (T_N_, CCR7^+^CD45RA^+^), central memory T cells (T_CM_, CCR7^+^CD45RA^-^), effector memory T cells (T_EM_, CCR7^-^CD45RA^-^) and terminally differentiated effector cells (T_EMRA_, CCR7^-^CD45RA^+^). Consist with previous studies, the frequencies of CD4^+^ and CD8^+^ T_N_ cells were remarkably decreased with age, along with a dramatic increase in the frequencies of CD4^+^ T_CM_ cells or CD8^+^ T_CM_, T_EM_ and T_EMRA_ cells (Supplementary Figure 1). The T_CM_, T_EM_, and T_EMRA_ subsets of both CD4^+^ and CD8^+^ T cells, known as antigen-experienced T cells, expressed higher levels of CD70 than T_N_ cells regardless of age (Figure 2). Also, CD70 expression was substantially increased in each T cell subset of CD4^+^ and CD8^+^ cells from older subjects as compared to young and middle-aged subjects (Figure 2). Thus, an elevated proportion of CD70^+^ fractions among CD4^+^ and CD8^+^ cells in elderly individuals was not only a result of the higher number of antigen-encountered T cells, but also the age-related increase of CD70 expression. Collectively, these results showed that CD70 up-regulation is a common characteristic of T cell immunosenescence.

### Aged CD70^+^ T cells displayed a phenotype of over-activation and exhaustion

In order to characterize the phenotype of CD70^+^ T cells in elderly individuals, we examined the expression levels of multiple activation markers, co-stimulatory and co-inhibitory molecules on the CD70^+^ and CD70^-^ fractions of T cells.

**Figure 3:**
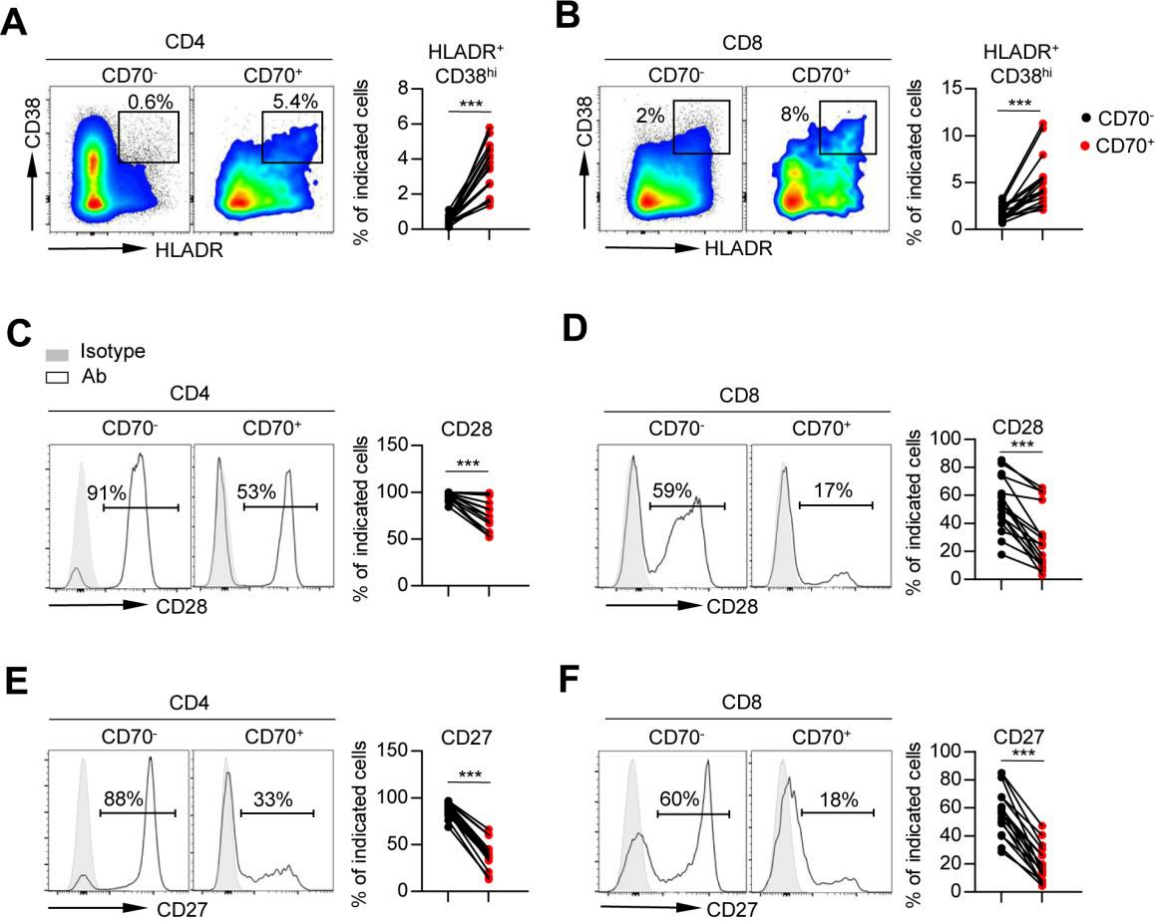
**Aged CD70^+^ T cells show a phenotype of over activation.** Flow cytometry analysis of percentage of HLA-DR^+^ CD38^hi^ cells (**A**–**B**), and expression of CD28 (**C**–**D**) and CD27 (**E**–**F**) on CD70^-^ vs. CD70^+^ CD4^+^ and CD8^+^ T cells from elderly individuals (61-80 years, n = 17). Representative flow data or histograms (left) and plots (right) display the expression of the above receptors on CD70^-^ vs. CD70^+^ cells (gated with CD4^+^ or CD8^+^ T cells). The *p*-values were obtained by paired t-test (HLA-DR^+^CD38^hi^ [CD4^+^ T cells], CD28, CD27) or Wilcoxon matched-pairs signed rank test (HLA-DR^+^CD38^hi^ [CD8^+^ T cells]). ***p < 0.001.

**Figure 4:**
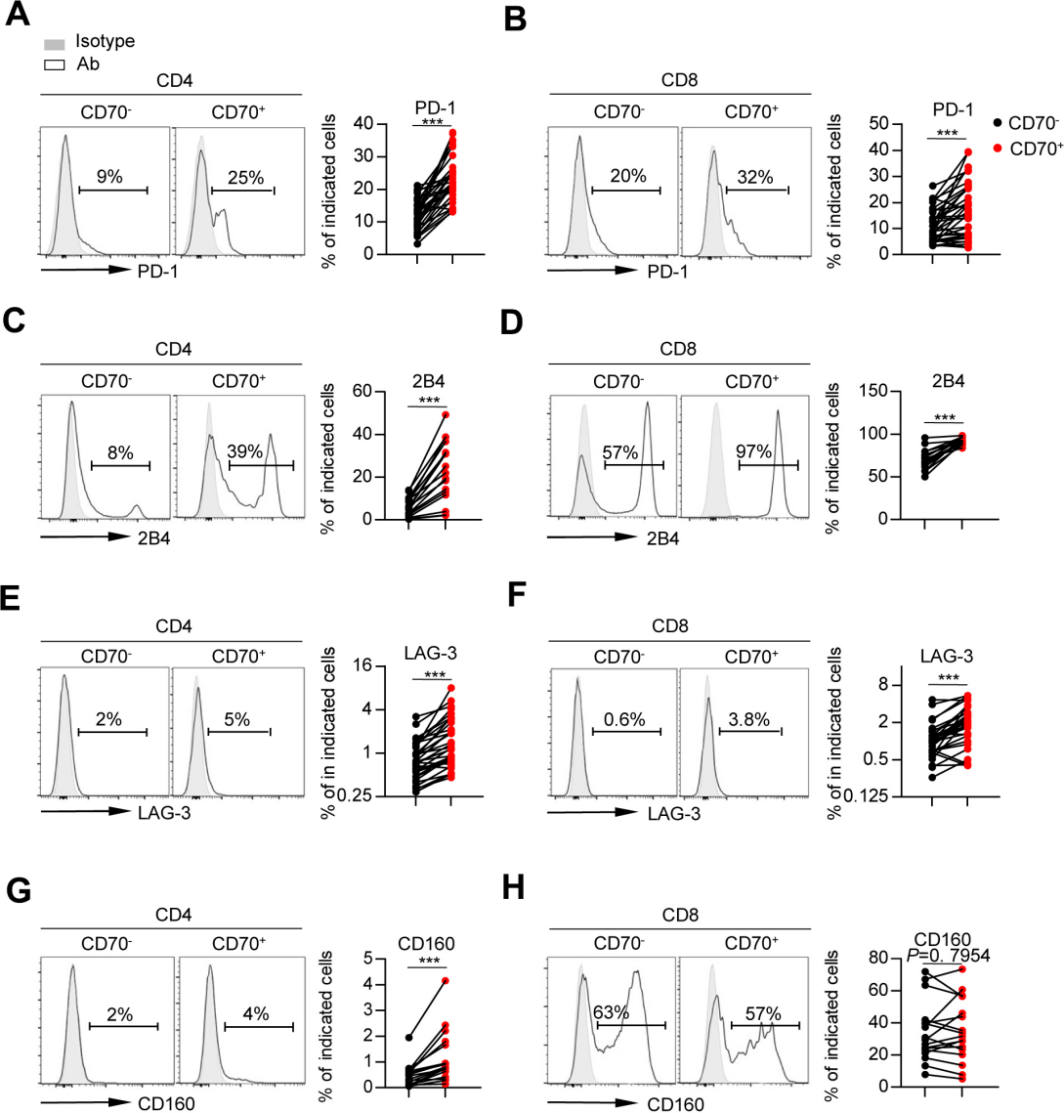
**CD70 expression is associated with the phenotypic profile of exhaustion.** Flow cytometry analysis of expression of PD-1 (**A**–**B**), 2B4 (**C**–**D**), LAG-3 (**E**–**F**) and CD160 (**G**–**H**) on CD70^-^ vs. CD70^+^ CD4^+^ and CD8^+^ T cells from elderly individuals (61-80 years, n = 17 [2B4, CD160], n = 34 [PD-1, LAG-3]). Representative histograms (left) and plots (right) display the expression of the above receptors on CD70^-^ vs. CD70^+^ cells (gated with CD4^+^ or CD8^+^ T cells). The *p*-values were obtained by paired t-test (PD-1 [CD4^+^ T cells], 2B4, CD160 [CD8^+^ T cells]) or Wilcoxon matched-pairs signed rank test (PD-1 [CD8^+^ T cells], LAG-3, CD160 [CD4^+^ T cells]). ***p < 0.001.

First, we assessed the frequency of activated T cells by detecting the co-expression of HLA-DR and CD38. The data showed a higher percentage of HLA-DR^+^ CD38^hi^ T cells in the CD70^+^ fraction than in CD70^-^ T cells regardless of age (Figure 3A, 3B and Supplementary Figure 2A). Analysis of the entire cohort demonstrated a close correlation between CD70 expression and the frequencies of activated CD4^+^ and CD8^+^ T-cells (Supplementary Figure 2D). Next, CD70^+^ T cells of all ages showed lower expression of co-stimulatory molecules CD28 and CD27 as compared to CD70^-^ fractions, indicating immune incompetence (Figure 3C–3F, Supplementary Figure 2B–2C), and expression of CD28 and CD27 on CD4^+^ T cells was negatively correlated with CD70 expression (Supplementary Figure 2E–2F). Finally, we observed significantly increased expressions of co-inhibitory molecules including PD-1, 2B4, and LAG-3 on CD70^+^ CD4^+^ and CD8^+^ T cells as compared to CD70^-^ T cells in elderly individuals (Figure 4A–4F). Moreover, higher level of CD160 expression was only found on CD70^+^ CD4^+^ T cells as compared to CD70^-^ subsets in elderly individuals (Figure 4G–4H). Similar results were observed in CD70^+^ T cells from the young and middle-aged individuals (Supplementary Figure 3A–3D). Additionally, expression of CD70 was strongly correlated with 2B4, CD160 or LAG-3 frequencies, but not with PD-1 frequencies (Supplementary Figure 3E–3H). In contrast, CD70^+^ T cells from elderly individuals expressed comparable levels of TIGIT and TIM-3 with CD70^-^ T cells (Supplementary Figure 4).

Overall, these results indicated an over-activated and consequently exhausted status of aged CD70^+^ T cells.

### CD70^+^ T cells in elderly individuals displayed increased sensitivity to apoptosis, which could be reversed by blocking CD70

**Figure 5:**
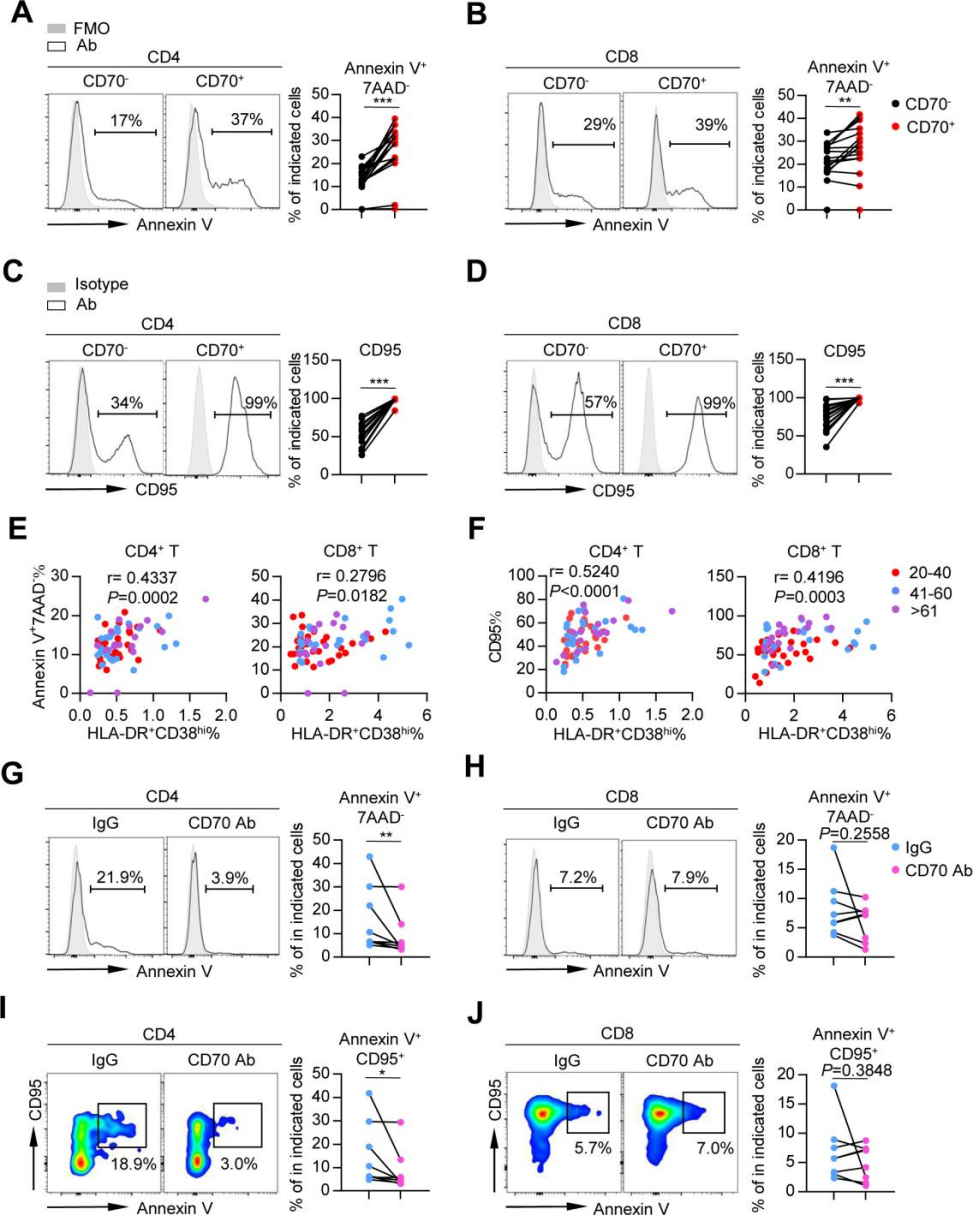
**Aged CD70^+^ T cells exhibit high susceptibility to apoptosis that can be reversed by blocking CD70.** (**A**–**D**) Percentage of apoptotic cells (Annexin V^+^ 7AAD^-^) (**A**–**B**) and expression of CD95 (**C**–**D**) in CD70^-^ and CD70^+^ T cells from elderly individuals (61-80 years, n = 17). Representative histograms (left) and plots (right) of the percentage of apoptotic cells are shown. The *p*-values were obtained by paired t-test (Annexin V) or Wilcoxon matched-pairs signed rank test (CD95). (**E**–**F**) Correlation analysis of percentage of HLA-DR^+^CD38^hi^ cells and percentage of Annexin V^+^ 7AAD^-^ cells (**E**) or CD95 expression (**F**) on CD4^+^ T cells (left) and CD8^+^ T cells (right) from all healthy donors. Spearman’s non-parametric test was used for correlation analysis. (**G**–**J**) Purified CD4^+^ and CD8^+^ T cells from elderly individuals (n = 8) were cultured *in vitro* with anti-human CD70 antibody or isotype IgG at a concentration of 10 μg/mL. After culturing for 24 h, the susceptibility to apoptosis was evaluated by flow cytometry. Representative histogram (left) and plot (right) of percentage of Annexin V^+^ 7AAD^-^ (**G**–**H**) and Annexin V^+^ CD95^+^ cells (**I**–**J**) in CD4^+^ and CD8^+^ T cells. The *p*-values were obtained by paired t-test (Annexin V^+^ 7AAD^-^ [CD8^+^ T cells], Annexin V^+^ CD95^+^ [CD8^+^ T cells]) or Wilcoxon matched-pairs signed rank test (Annexin V^+^ 7AAD^-^ [CD4^+^ T cells], Annexin V^+^ CD95^+^ [CD4^+^ T cells]). *p < 0.05, **p < 0.01, ***p < 0.001.

To assess the functional status of CD70^+^ T cells from the elderly, we detected susceptibility of these cells to apoptosis by measuring percentage of apoptotic cells (Annexin V^+^ 7AAD^-^) and expression of CD95 (Fas). Percentage of apoptotic cells and CD95 expression were significantly elevated on CD70^+^ CD4^+^ and CD8^+^ T cells (Figure 5A–5D and Supplementary Figure 5A, 5B), suggesting a high susceptibility to apoptosis. These results were confirmed by the close correlation between CD70 and percentage of Annexin V^+^ 7AAD^-^ cells in CD4^+^ T cells (r = 0.63982, *p* = 0.0006; Supplementary Figure 5C), as well as CD95 expression in both CD4^+^ (r = 0.6026, *p* < 0.0001) and CD8^+^ T cells (r = 0.4535, *p* < 0.0001; Supplementary Figure 5D). Of note, percentage of Annexin V^+^ 7AAD^-^ cells and CD95 expression in T cells were strongly correlated with the percentage of activated HLA-DR^+^ CD38^hi^ T cells, implying the role of activation-induced cell death (AICD) in CD70 associated T cell aging (Figure 5E and 5F).

The specific role of CD70 in the induction of T-cell apoptosis was examined by blocking CD70 using an anti-CD70 neutralizing antibody. In the presence of the neutralizing antibody, percentage of Annexin V^+^ 7AAD^-^ cells and CD95 expression were decreased in aged CD4^+^ T cells (Figure 5G–5J). This data indicated the important suppressive role of CD70 in the regulation of T-cell function in elderly individuals.

### Aged CD70^+^ T cells showed increased levels of inflammatory cytokines and intracellular proteins

**Figure 6:**
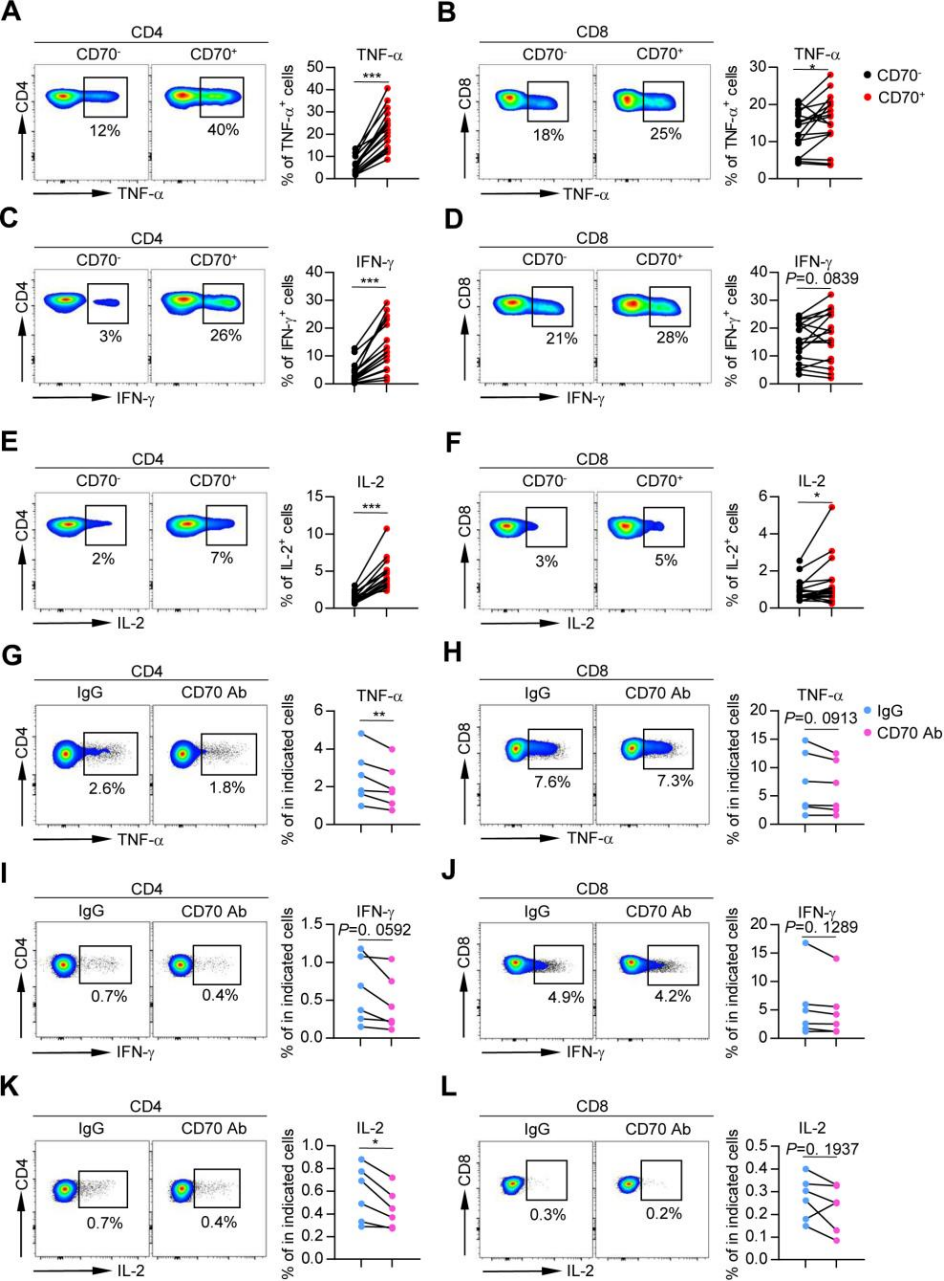
**Aged CD70^+^ T cells secrete increased levels of inflammatory cytokines that can be reversed by blocking CD70.** (**A**–**F**) Intracellular staining for TNF-α, IFN-γ, and IL-2 in CD70^-^ and CD70^+^ T cells from elderly individuals (61-80 years, n = 17) after *in vitro* anti-CD3/anti-CD28 stimulation. Representative flow data (left) and plots (right) for TNF-α, IFN-γ, and IL-2, respectively. The *p*-values were obtained by paired t-test (TNF-α, IFN-γ [CD8^+^ T cells], IL-2 [CD4^+^ T cells]) or Wilcoxon matched-pairs signed rank test (IFN-γ [CD4^+^ T cells], IL-2 [CD8^+^ T cells]). (**G**–**L**) Purified CD4^+^ and CD8^+^ T cells from healthy individuals (n = 6) were cultured with antagonist anti-CD70 antibody or IgG at a concentration of 10 μg/mL as indicated. After culturing *in vitro* for 24 h and stimulating with anti-CD3/anti-CD28, the cytokine production was measured by flow cytometry. Representative histogram (left) and plot (right) of TNF-α, IFN-γ, and IL-2 expression in CD4^+^ and CD8^+^ T cells. The *p*-values were obtained by paired t-test. *p < 0.05, **p < 0.01, ***p < 0.001.

Senescent cells can secrete numerous inflammatory cytokines and chemokines, which act together to generate a proinflammatory environment [7, 24]. To determine whether CD70 is involved in senescence-associated inflammatory responses in T cells, we tested cytokine release after *in vitro* stimulation with anti-CD3 and anti-CD28. The results showed significantly increased levels of TNF-α, IFN-γ, and IL-2 in CD70^+^ CD4^+^ T cells as compared to CD70^-^ CD4^+^ T cells (Figure 6A, 6C, 6E). Slight elevations of these cytokines were observed in CD70^+^ CD8^+^ T cells from elderly individuals (Figure 6B, 6D, 6F). Similar results were observed in CD70^+^ CD4^+^ and CD8^+^ T cells from the young and middle-aged groups (Supplementary Figure 6A–6C). Importantly, TNF- α and IL-2 secretion was significantly decreased in aged CD4^+^ T cells after CD70 blocking (Figure 6G–6L). Moreover, CD70^+^ CD8^+^ T cells from all age groups showed elevated expression of perforin and Granzyme B, suggesting a greater non-specific killing potential. Interestingly, increased levels of perforin and Granzyme B were also observed in CD70^+^ CD4^+^ T cells (Supplementary Figure 6D and 6E). Additionally, CD70^+^ T cells exhibited significantly higher levels of Ki-67 than CD70^-^ T cells regardless of age (Supplementary Figure 6F).

Taken together, these results indicated that aged CD70^+^ T cells produced high levels of inflammatory cytokines and intracellular Granzyme B and perforin.

## DISCUSSION

It has been demonstrated that down-regulation of some co-stimulatory molecules and up-regulation of some co-inhibitory molecules are key features of T cell aging. However, this study showed a significant age-related accumulation of CD70, which was generally regarded as a co-stimulatory molecule, on both CD4^+^ and CD8^+^ T cells. Consistent with its contradictory role in T cell activation, which was reported by recent studies, the findings of this study highlighted an important role of CD70 in T cell aging. CD70^+^ T cells from elderly individuals displayed phenotypic features of exhaustion and high susceptibility to apoptosis. In contrast, aged CD70^+^ T cells also produced higher levels of pro-inflammatory cytokines and expressed more intracellular Granzyme B and perforin, which was consistent with an important feature of senescent cells known as the senescence-associated secretory phenotype (SASP) [24, 25]. These data indicated that CD70 is a biomarker of T cell aging and elucidated a potential mechanism of aging. To the best of our knowledge, this is the first evidence for the involvement of CD70 in immunosenescence.

Since aged CD70^+^ T cells expressed numerous co-inhibitory molecules, suggesting a phenotype of exhaustion, we compared senescent and exhausted T cells. Both cells were dysfunctional in some aspects. However, they differed from each other in inflammatory cytokine and intracellular protein expression. Recent studies indicated that senescent cells are metabolically active rather than dormant. They expressed numerous cytokines, chemokines, growth factors, and proteases, which was characterized as SASP [24, 26]. However, previous studies including ours indicated that up-regulation of co-inhibitory molecules such as PD-1, TIM-3, or TIGIT on aged T cells induced defective cytokine production, suggesting exhaustion rather than senescence [10, 14, 27]. In the present study, CD70^+^ T cells from elderly individuals showed increased pro-inflammatory cytokines such as TNF- α and IFN- γ, and higher levels of intracellular Granzyme B and perforin. These findings supported the notion that senescence was associated with enhanced chronic inflammation. Notably, once chronic inflammation was established, it often induced the immune system to produce more cytokines through positive feedback loops [25]. Thus, it was thought to underlie the increased incidence of autoimmune diseases in the elderly. Previous studies reported that CD70 was overexpressed on human proinflammatory Th1 and Th17 cells, which contributed to pathogenesis of multiple sclerosis [28]. Increased CD70^+^ CD4^+^ T lymphocytes were also observed in systemic lupus erythematosus (SLE) and rheumatoid arthritis (RA) patients [29–31], further confirming the hypothesis.

More importantly, increased inflammatory cytokines from aged CD70^+^ T cells induced a persistent over-activated status that finally led to apoptosis. It was characterized as AICD, which might account for CD70-associated immunosenescence. AICD is a critical pre-programmed death pathway that plays a central role in the aging process [32, 33]. Higher AICD rate was observed during replicative senescence *in vitro*, and was more pronounced in T cells from the elderly than young individuals [34]. A previous study showed that chronic immune stimulation induced overexpression of death receptors on aged T cells, leading to up-regulation of AICD [35, 36]. Cultured PBMCs from elderly individuals expressed higher CD95 levels than those from young individuals upon activation [34, 37, 38]. Consistently, the present study showed an increased activated HLA-DR^+^ CD38^hi^ population as well as up-regulation of percentage of apoptotic cells and CD95 expression on aged CD70^+^ T cells. Moreover, blocking CD70 decreased apoptosis levels in aged CD4^+^ T cells. These results were further confirmed by close correlation between percentage of apoptotic cells and CD95 expression, and percentage of activated HLA-DR^+^ CD38^hi^ T cells.

Several surface markers were reported to track the “age” of human circulating T cells, such as loss of CD27 and CD28, or gain of CD57 and killer cell lectin-like receptor sub family G (KLRG-1) [10, 39–41]. CD4^+^ and CD8^+^ T cells share some phenotypic changes during aging, however, age-related changes occur more frequently in CD8^+^ T cells than in CD4^+^ T cells. For instance, CD8^+^ T cells lost CD28 more rapidly than CD4^+^ cells during aging [42]. CD57^+^ CD8^+^ T cells and KLRG1^+^ CD8^+^ T cells were recruited with age [43, 44]. Our previous study showed that TIGIT contributed to CD8^+^ T cell aging. However, CD4^+^ T cells were less sensitive to age, with a greater homeostatic stability when compared to CD8^+^ T cells [40, 45]. The present study showed that CD70 was involved in aging of both CD4^+^ and CD8^+^ T cells. Of note, CD4^+^ T cells exhibited more significant up-regulation of CD70 during aging than CD8^+^ T cells. Collectively, these data suggested CD70 as a biomarker associated with T cell aging, especially for CD4^+^ T cells.

In summary, the present study demonstrated CD70 as a prominent regulator involved in immunosenescence, which led to defects and overwhelming inflammatory responses of T cells during aging. These findings may be beneficial in the treatment of age-related comorbidities.

## MATERIALS AND METHODS

### Participants

This study was approved by the Committee of Ethics at Beijing Ditan Hospital, Capital Medical University, Beijing, China. All participants were healthy volunteers aged 18-80 years (94 men and 123 women) who were recruited between February 2016 and October 2017. Gender was evenly distributed in each group. Participants who tested positive to human immunodeficiency virus (HIV) infection, hepatitis viral infections, systemic infection, connective tissue disease, cancer or abnormal tumor markers, including alpha fetoprotein (AFP), carcinoembryonic antigen (CEA), carbohydrate antigen (CA-199), CA-153, and CA-125, were excluded.

### Isolation of peripheral blood mononuclear cells (PBMCs)

Peripheral blood samples were collected from healthy donors and PBMCs were purified using standard Ficoll-Paque gradient centrifugation according to the manufacturer’s instructions (Amersham Pharmacia Biotech, Sweden). Cells were cryopreserved in fetal bovine serum (GIBCO, Grand Island, NY, USA) supplemented with 10% DMSO, and stored in liquid nitrogen.

### Immunofluorescence staining and flow cytometry analysis

PBMCs were incubated with directly conjugated antibodies for 30 min at 4°C. The cells were washed before flow cytometry analysis. Antibodies used included anti-human CD3-BV786, CD4-APC-Fire750, CD8-BV510, CD45RA-AF700, CD70-PE, PD-1-BV711, 2B4-FITC, CD160-AF488, TIM-3-BV650, CD95-PE-CY7 (BD Biosciences, San Diego, CA, USA), CCR7-BV421, HLA-DR-AF700, CD38-BV421, CD28-BV711, CD27-BV650 (BioLegend, San Diego, CA, USA), TIGIT-PE-Cy7, LAG-3-APC (Ebioscience, San Diego, CA, USA) and the corresponding isotype controls. Data acquisition was performed on an LSR Fortessa flow cytometer (BD Biosciences), and data was analyzed with FlowJo software (Tree Star, Ashland, OR, USA).

### *In vitro* stimulation and intracellular staining

PBMCs were cultured in RPMI-1640 medium (GIBCO, Grand Island, NY, USA) containing 10% FBS, and stimulated with anti-CD3/CD28 (2 μg/mL and 5 μg/mL, Ebioscience) plus Golgiplug (BD Biosciences) for 5 h. The cells were surface-stained with CD3-BV786, CD4-APC-Fire750, CD8-BV421, CD70-PE, and intracellularly stained with TNF-α-BV711 (BD Biosciences), IFN-γ-AF700 (Ebioscience), or IL-2-BV650 (BioLegend) antibodies. For Ki67, perforin or Granzyme B staining, PBMCs were surface-stained with CD3-BV786, CD4-APC-Fire750, CD8-BV421, CD70-PE, and intracellularly stained with Granzyme B-AF700 (BD Biosciences), Ki67-BV711, or perforin-APC (BioLegend) antibodies. A fixable viability dye eFluor® 506 (Ebioscience) was used to assess cell viability.

### Analysis of T-cell apoptosis

Apoptosis rates were measured using an APC Annexin V apoptosis detection kit (BioLegend) as per the manufacturer’s instructions, in combination with markers for T cells. Samples were analyzed by flow cytometry.

### Cell separation and CD70 blockage

CD4^+^ and CD8^+^ T cells were isolated from PBMCs by positive selection using EasySep™ human CD4 and CD8 positive selection kit (StemCell Technologies, Vancouver, Canada). Purified cells were cultured at a concentration of 1 × 10^6^ cells/mL in a 96 well tissue culture plate and 10 μg/ml anti-human CD70 antibody (clone 113-16; BioLegend) or isotype control was added to the culture medium. After 24 h of culture, Annexin V staining and cytokine production were measured by flow cytometry.

### Statistical analysis

The data are expressed as the mean ± standard deviation (SD). GraphPad7 (GraphPad Software, La Jolla, CA, USA) or SPSS (IBM Corporation, New York, NY, USA) were used for statistical analyses. The normality of each variable was evaluated using the Kolmogorov-Smirnov test. For normally distributed data, the comparison of two variables was performed using unpaired, or paired where specified, two-tailed Student’s t-tests for unpaired and paired data, respectively. One-way ANOVA followed by Tukey’s multiple comparisons test was used for comparing two or more independent samples. When the data were not normally distributed, the comparison of variables was performed with a Mann-Whitney U test or a Wilcoxon matched-pairs signed rank test for unpaired and paired data, respectively. For comparing two or more independent samples, a Kruskal-Wallis test followed by Dunn’s multiple comparisons test was used. Participant characteristics were compared using Chi-square test (categorical variables) or Kruskal-Wallis test (continuous variables). Pearson’s or Spearman’s correlation coefficients were used to evaluate correlations for normally or non-normally distributed data, respectively. For all analyses, *p*-values <0.05 were considered statistically significant.

## Supplementary Material

Supplementary Figures
