# Supplementary material for: CD70 contributes to age-associated T cell defects and overwhelming inflammatory responses
Source: Aging (Albany NY). 2020 Jun 19;12(12):12032–50. doi: 10.18632/aging.103368 (PMC7343466; doi:10.18632/aging.103368)
Supplement: Supplementary Figures [file aging-12-103368-s001..pdf]

SUPPLEMENTARY FIGURES

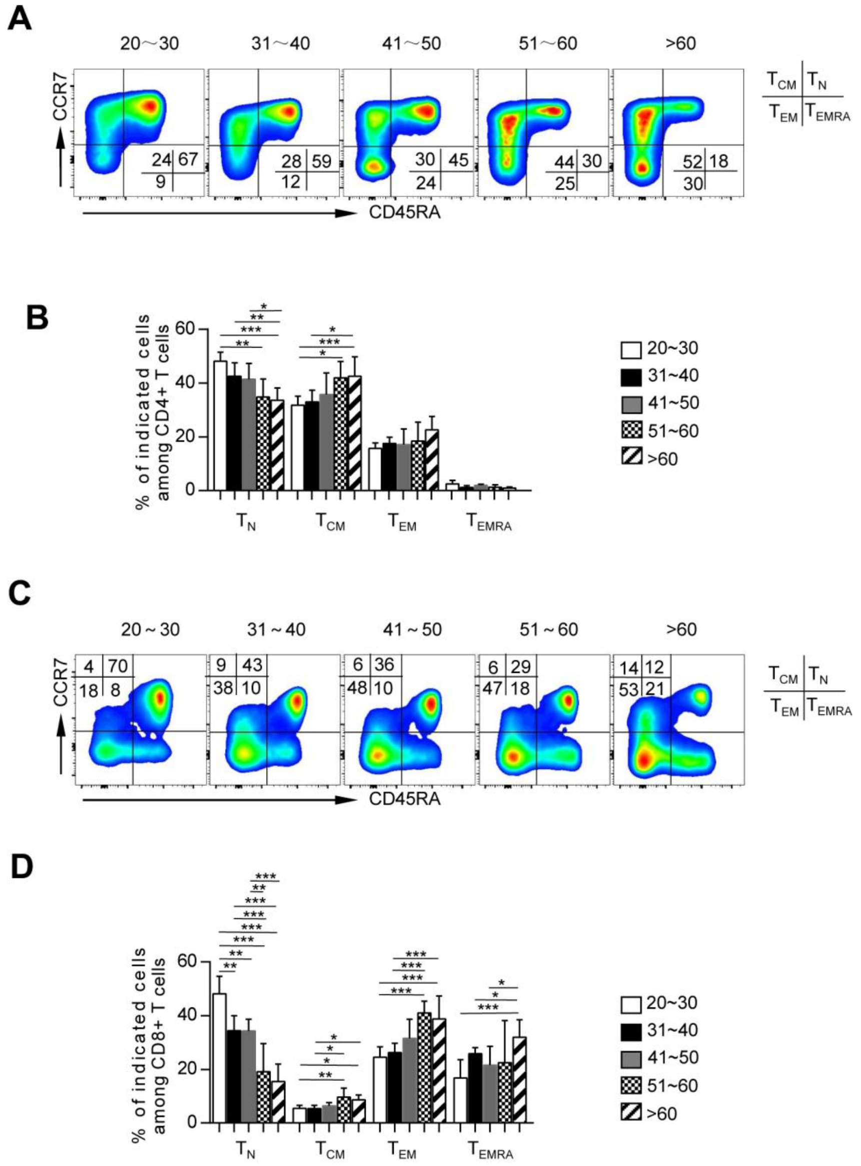

Supplementary Figure 1. Distribution of $CD4^+$ and $CD8^+$ T cell subsets from different age groups. Distribution of T_N, T_{CM}, T_{EM}, and T_{EMRA} in $CD4^+$ and $CD8^+$ T cells from different age groups. Representative flow data (**A**, **C**) and box plots (**B**, **D**) of the percentage of each subset in different age groups are shown (n = 34-56 each group). Data are shown as the median ± 95% confidence interval (CI). The *p*-values were obtained by Kruskal-Wallis test followed by Dunn's multiple comparisons test [T_{CM}, T_{EM} ($CD4^+$ T cells), T_{EMRA}] or one-way ANOVA test followed by Tukey's multiple comparisons test [T_N, T_{EM} ($CD8^+$ T cells)]. *$p < 0.05$, **$p < 0.01$, ***$p < 0.001$.

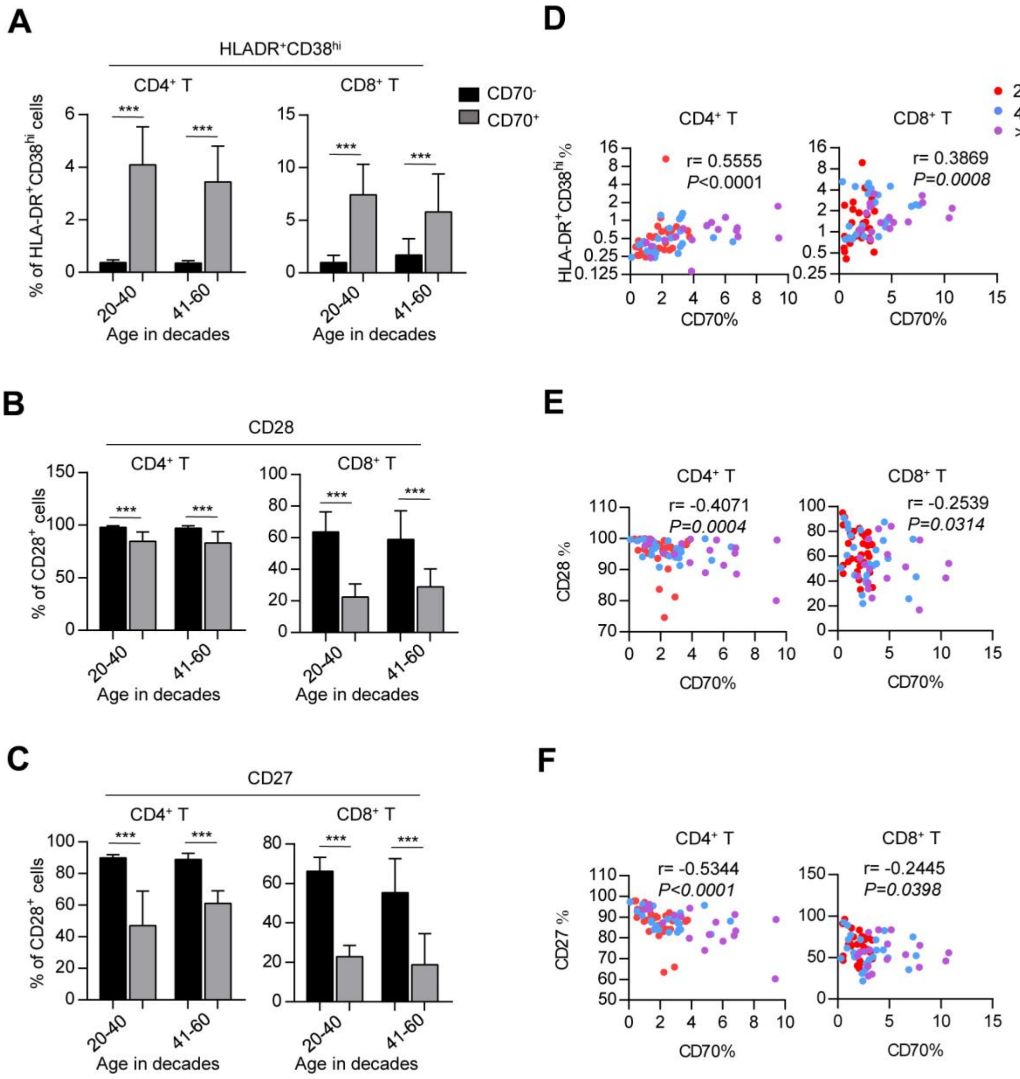

Supplementary Figure 2. Elevated immune activation and decreased co-stimulatory signaling on CD4$^+$ and CD8$^+$CD70$^+$ T cells. (**A**–**C**) Flow cytometry analysis of the percentage of HLA-DR$^+$CD38^{hi} cells (**A**), expression of CD28 (**B**) and CD27 (**C**) on CD70$^-$ vs. CD70$^+$CD4$^+$ and CD8$^+$ T cells from young and middle-aged adults (21-40 years old for young, n = 31; 41-60 years old for middle-aged, n = 24). Each bar represents median ± 95% confidence interval, CI. The *p*-values were obtained by Kruskal–Wallis test followed by Dunn's multiple comparisons test. (**D**–**F**) Correlation analysis of CD70 and percentage of HLA-DR$^+$CD38^{hi} cells (**D**), expression of CD28 (**E**) and CD27 (**F**) on CD4$^+$ T cells (left) and CD8$^+$ T cells (right). Spearman's non-parametric test was used for correlation analysis. ***$p < 0.001$.

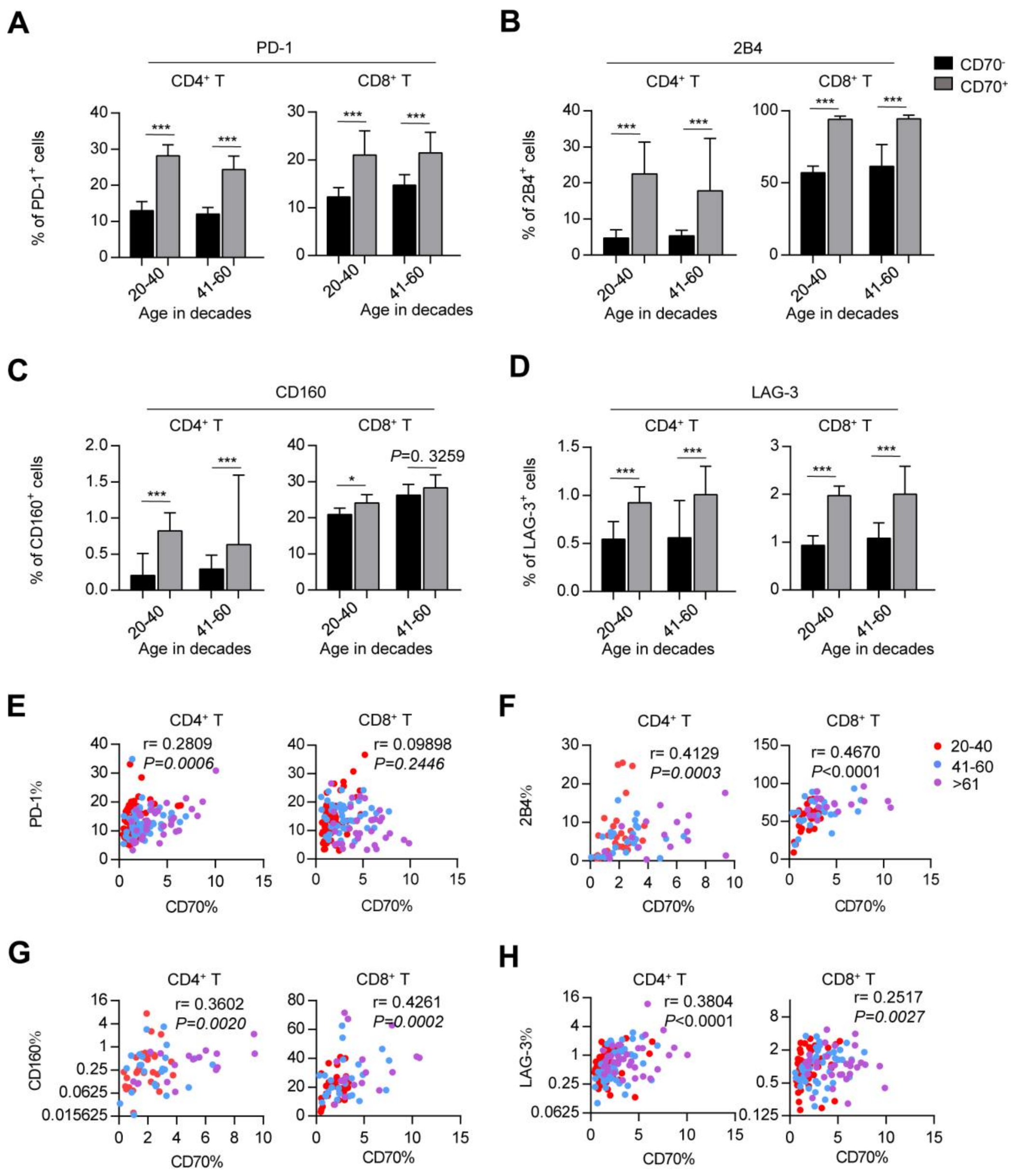

Supplementary Figure 3. CD70 expression is associated with certain inhibitory receptors on $CD4^+$ and $CD8^+$ T cells. (**A**–**D**) Flow cytometry analysis of the expression of PD-1 (**A**), 2B4 (**B**), CD160 (**C**) and LAG-3 (**D**) on $CD70^-$ vs. $CD70^+$ $CD4^+$ and $CD8^+$ T cells from young and middle-aged groups (n = 24-63 each group). Data are represented as median ± 95%CI [PD-1, 2B4, CD160 ($CD4^+$ T cells), LAG-3] or mean ± SEM [CD160 ($CD8^+$ T cells)]. The *p*-values were obtained by Kruskal–Wallis test followed by Dunn's multiple comparisons test [PD-1, 2B4, CD160 ($CD4^+$ T cells), LAG-3] or one-way ANOVA test followed by Tukey's multiple comparisons test [CD160 ($CD8^+$ T cells)]. (**E**–**H**) Correlation analysis of CD70 and expression of PD-1 (**E**), 2B4 (**F**), CD160 (**G**) and LAG-3 (**H**). Spearman's non-parametric test was used to test for correlations. * p <0.05, *** p < 0.001.

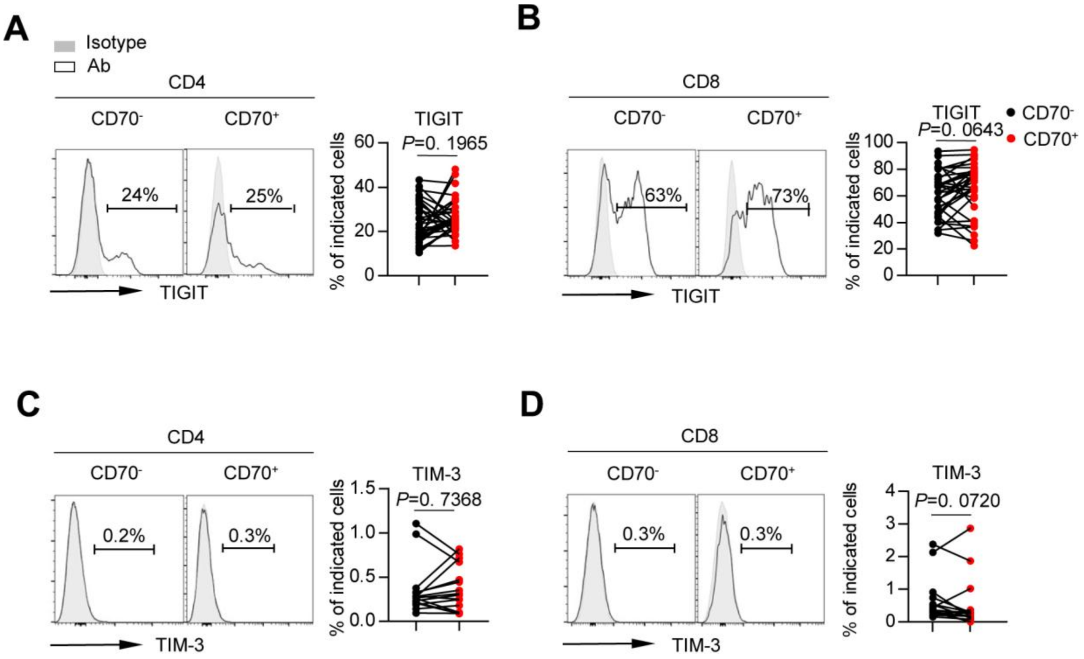

Supplementary Figure 4. Expression levels of TIGIT and TIM-3 on CD70$^-$ and CD70$^+$ T cells from elderly individuals. Flow cytometry analysis of the expression of TIGIT (**A**–**B**) and TIM-3 (**C**–**D**) on CD70$^-$ vs. CD70$^+$CD4$^+$ and CD8$^+$ T cells from the elderly (61–80 years old, n = 34 [TIGIT], n = 17 [TIM-3]). Representative histograms (left) and plots (right) display the expression of the above receptors on CD70$^-$ vs. CD70$^+$ cells. The *p*-values were obtained by Wilcoxon matched-pairs signed rank test.

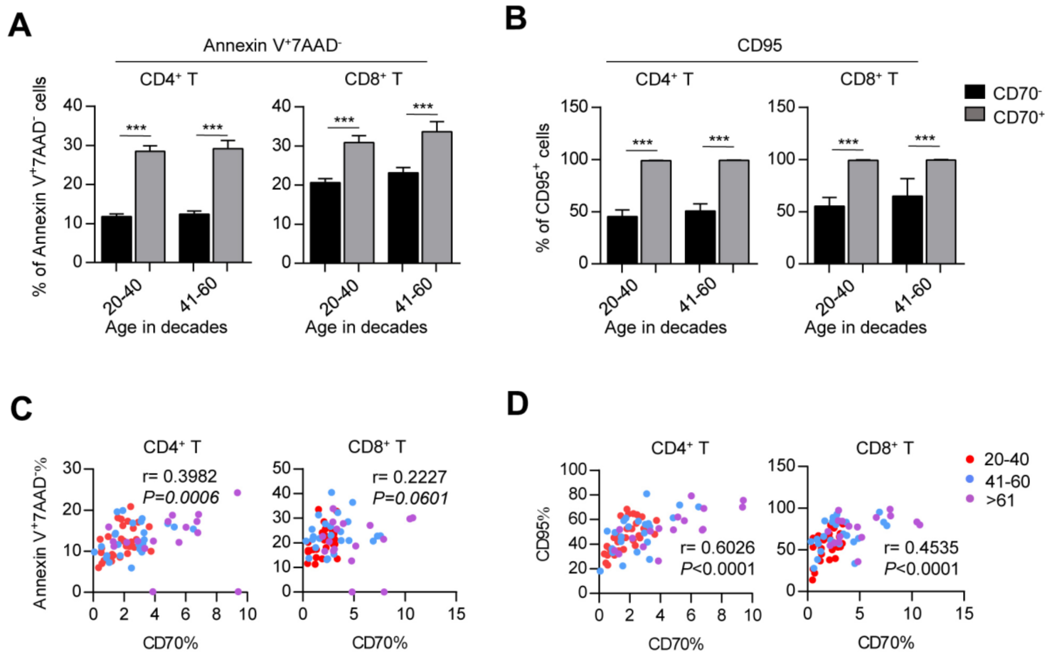

Supplementary Figure 5. CD70$^+$ T cells from different age groups exhibit high susceptibility to apoptosis. (**A**–**B**) Flow cytometry analysis of percentage of apoptotic cells (Annexin V$^+$ 7AAD$^-$) (**A**) and expression of CD95 (**B**) in CD70$^-$ and CD70$^+$ T cells from young and middle-aged adults ((21-40 years old for young, n = 31; 41-60 years old for middle-aged, n = 24). Data are represented as mean ± SEM (Annexin V$^+$ 7AAD$^-$) or median ± 95%CI (CD95). The *p*-values were obtained by Kruskal–Wallis test followed by Dunn's multiple comparisons test (CD95) or one-way ANOVA test followed by Tukey's multiple comparisons test (Annexin V$^+$ 7AAD$^-$). (**C**–**D**) Correlation analysis of CD70 and percentage of Annexin V$^+$ 7AAD$^-$ cells (**C**) or CD95 expression (**D**) on CD4$^+$ T cells (left) and CD8$^+$ T cells (right). Spearman's non-parametric test was used for correlation analysis. *** $p < 0.001$.

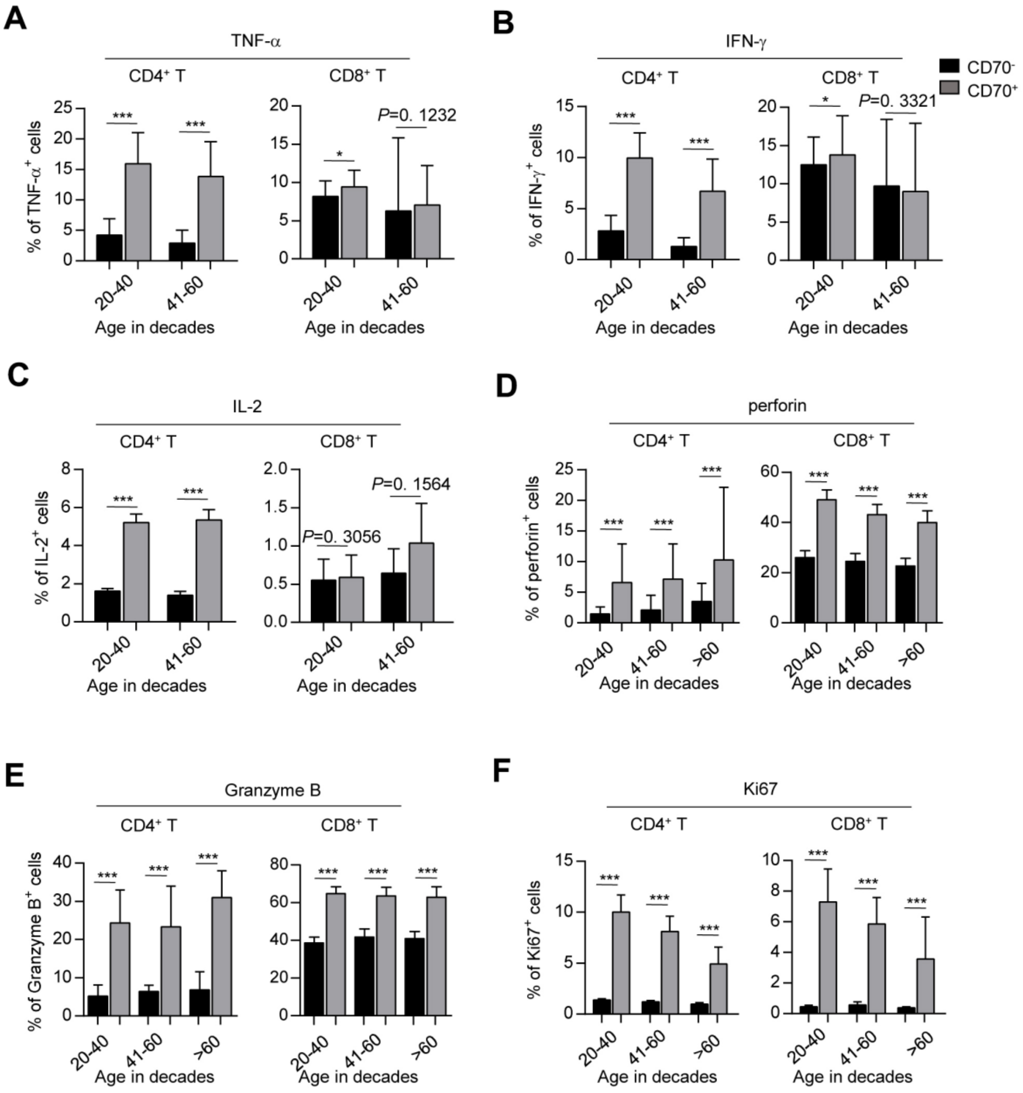

Supplementary Figure 6. CD70$^+$CD4$^+$ T cells from different age groups exhibit increased levels of inflammatory cytokines, while CD70$^+$CD4$^+$ and CD8$^+$ T cells show increased proliferation and cytotoxicity. (**A**–**C**) Intracellular staining for TNF-α (**A**), IFN-γ (**B**), and IL-2 (**C**) on CD70$^-$ vs. CD70$^+$CD4$^+$ and CD8$^+$ T cells from young and middle-aged adults (21-40 years old for young, n = 24; 41-60 years old for middle-aged, n = 19) upon in vitro anti-CD3/anti-CD28 stimulation. (**D**–**F**) Expression of perforin (**A**), Granzyme B (**B**) and Ki-67 (**F**) in CD70$^-$ and CD70$^+$ T cells from different age groups (n = 17-31 each group). Data are shown as mean ± SEM [IL-2 (CD4$^+$ T cells), perforin (CD8$^+$ T cells), Granzyme B (CD8$^+$ T cells)] or median ± 95%CI [TNF-α, IFN-γ, IL-2 (CD8$^+$ T cells), perforin (CD4$^+$ T cells), Granzyme B (CD4$^+$ T cells), Ki-67]. The *p*-values were obtained by Kruskal–Wallis test followed by Dunn's multiple comparisons test [TNF-α, IFN-γ, IL-2 (CD8$^+$ T cells), perforin (CD4$^+$ T cells), Granzyme B (CD4$^+$ T cells), Ki-67] or one-way ANOVA test followed by Tukey's multiple comparisons test [IL-2 (CD4$^+$ T cells), perforin (CD8$^+$ T cells), Granzyme B (CD8$^+$ T cells)]. * $p < 0.05$, *** $p < 0.001$.
